# How Can Doctors Help Their Patients to Return to Work?

**DOI:** 10.1371/journal.pmed.0030088

**Published:** 2006-03-28

**Authors:** Jos H Verbeek

## Abstract

Getting back to work is important for most patients but doctors are often unsure how best to help. The article reviews evidence for the effectiveness of the interventions now available.

For most patients, work remains an important part of life. However, patients with chronic diseases often encounter difficulties upon returning to work after an episode of illness [ 1]. Helping to reintegrate patients into their workplace should, therefore, be an important treatment goal for every doctor. However, in a recent review of back pain treatment, we found that patients were dissatisfied because they did not get practical instructions from their physicians on how to cope with everyday problems [ 2]. The same was found in a qualitative study of patients with breast cancer [ 3]. When we asked cancer survivors if they had discussed return to work with their attending physician, it turned out that only half of them had done so [ 4]. A reason for the perceived lack of support of patients might be that most doctors who treat patients feel unsure *how* they could be involved in “return-to-work issues” [ 5]. I would, therefore, like to provide a review of the most important theories involved in return to work and of the evidence for the effectiveness of interventions that improve a patient's functioning, including his or her return to work after an episode of illness.

## Opportunities for Interventions in the World Health Organization Model of Functioning

**Figure 1:**
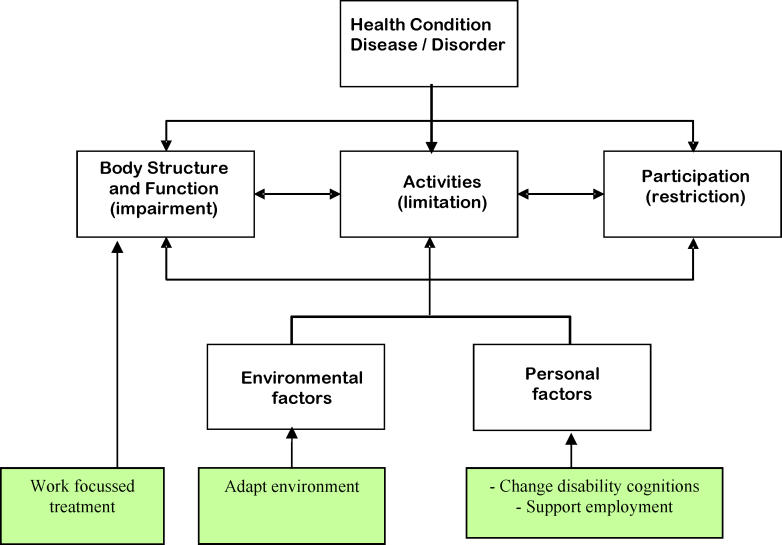
The WHO Model of Functioning, Disability, and Health

There is a wide range of disability among patients, even when they have had the same disease with equal severity. For example, among patients who have survived breast cancer after surgery and chemotherapy, sick leave is on average about a year, but it nevertheless varies from a couple of days to a couple of years. Among patients with testicular cancer who have undergone surgery, there is a variation of several weeks in the duration of sick leave [ 6]. For patients with nonspecific back pain, the variation is in a range of months [ 7]. It is not always easy to explain such variations, but the World Health Organization (WHO) provides a useful framework that helps in understanding the problem of return to work. The WHO explains in its International Classification of Functioning, Disability, and Health how disease and disability are related ( Figure 1). The model considers the influence of disease and its intermediaries on an individual's participation in society. Diseases or disorders affect the triad of “body structure and function”, “activities”, and “participation”, which lead to either disability or no disability depending on important conditional factors of environmental origin, such as heavy physical work, and of personal origin, such as personal ideas about disability [ 8].

The model offers three opportunities for intervention. The first opportunity is better treatment. In the 1970s and 1980s, a change in the treatment of heart disease greatly influenced its related disability [ 9]. It has also been argued that when work issues are addressed as part of treatment, return to work is more successful [ 10]. Secondly, the environmental factors provide an opportunity for intervention. The science of ergonomics has evolved around the concept of adapting the environment to workers [ 11]. This provides a strong incentive for occupational physicians to advocate workplace adaptations to prevent disability. Usually, these interventions are beyond the scope of clinicians. However, there is evidence that special arrangements made by the employer such as gradual return to work, which all doctors can recommend, facilitate the return to work of patients in general [ 12]. Thirdly, opportunities are provided by person-related factors; improving skills or learning new skills have been the focus of rehabilitation for a long time, especially for people with serious mental health problems. A Cochrane review shows, however, that supported employment is more effective than prevocational training [ 13]. Supported employment emphasizes rapid job placement for the patient and ongoing support after placement.

The WHO International Classification of Functioning model is supported by many studies that have investigated the prognosis for return to work among patients suffering from a variety of diseases. From these studies, it can be concluded that the severity of the disease resulting in impairment of body function or structure usually has the biggest influence on the time needed to return to work, but environmental factors and person-related factors play an additional role [ 6]. Looking further into the personal factors, it has been found that, for a wide variety of diseases, the expectations of the patient about recovery best predict the time taken to return to work [ 14]. The patient's prediction is better than that of the doctor [ 15].

## Personal Factors Explained by Behavioural Theories

Several theories explain the mechanisms of how the person-related factors mentioned in the WHO International Classification of Functioning model are important in predicting return to work. First, there is the well-known “theory of illness behaviour”, elaborated by David Mechanic, which states that people interpret bodily symptoms differently and as a consequence will behave differently [ 16]. It explains why, for example, some patients with back pain interpret their symptoms in such a way that it does not help their recovery. Among these patients, nonspecific back pain leads easily to a fear of movement. In turn, lack of functioning leads to longer disability, which reinforces the idea that there is something wrong with the back [ 17].

How our ideas about illness influence our way of coping with disease has been elaborated by E. A. Leventhal in the “model of illness representations”. The model, or theory, states that if the patient considers the disease as a narrowly defined medical disorder, the duration as long and the consequences as serious, the functional outcome will be worse, irrespective of the objective medical seriousness of the illness [ 18]. These ideas have also been called misconceptions about the disease. In patients with chronic fatigue syndrome, myocardial infarction, rheumatoid arthritis, and asthma, the patient's representation of the illness was strongly linked to the functional outcome [ 19–21]. Because this mechanism works over a range of diseases, this strongly suggests that the ideas a patient has about the disabilities that result from the illness are important in encouraging or hindering return to work. This could, therefore, provide an important opportunity for intervention by the clinician.

## Effective Return-to-Work Interventions

To find evaluations of return-to-work interventions, I searched Medline through PubMed with the search strategy recommended by Haafkens et al., i.e., combining the following text words with “or”: “return to work”, “returned to work”, “sick leave”, “sickness absence”, “work capacity”, “work disability”, “vocational rehabilitation”, absenteeism, retirement, “employment status”, and “work status”. I restricted the search to randomised controlled trials (RCTs), and to find psychological treatment, I added “psychol*” or “cognitive”. This yielded 225 studies. Of these, I found the following studies relevant.

For four major disease categories— heart disease, rheumatoid arthritis, back pain, and common mental health disorders—studies have been conducted to determine if these prognostic factors are amenable to change ( Table 1).

**Table 1:**
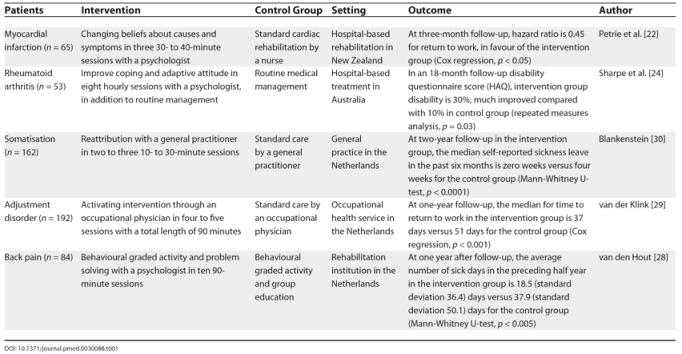
Person-Directed Interventions for Return to Work and Disability in Various Diseases Proven to Be Successful in Randomised Controlled Trials

In an RCT among hospital patients with myocardial infarction, perception of the illness was changed by a brief psychological intervention leading to a twice-as-fast rate of return to work [ 22]. The intervention used the patient's perceptions of their illness as a starting point. It was specifically structured to change highly negative misperceptions of the timeline for return to work and the consequences of myocardial infarction. This finding contrasts with the lack of effect on return-to-work measures of cardiac rehabilitation programmes that do not focus on psychological treatment [ 23]. In an RCT among patients with rheumatoid arthritis, cognitive-behavioural therapy has also been shown to improve joint function and self-reported daily functioning at and outside of work [ 24].

For nonspecific lower back pain, Gordon Waddell was one of the first to recognise the importance of a patient's beliefs about the disease and the social interactions between patients and the environment [ 25]. According to two Cochrane reviews, therapy with a behavioural component is an effective treatment in patients with chronic back pain, and is also capable of substantially reducing the number of sick days taken by these patients [ 26, 27]. For example, adding problem solving to the physical therapy of patients with back pain shortened sickness absence in a rehabilitation setting [ 28].

Lastly, patients with common mental health problems such as adjustment disorder, depression, anxiety, or somatisation problems are especially prone to long-term disability. Two cluster-randomised trials showed that sick leave can be reduced substantially among patients with common mental disorders. In one, a cognitive-behavioural approach of workers with adjustment disorder improved the rate of return to work in comparison with standard care [ 29]. The other trial was performed in general practice among somatising patients. In this trial, sickness absence decreased by several weeks among those who were treated according to a cognitive-behavioural model, compared with those who received standard care [ 30]. However, in one randomised trial, general practitioners were not able to shorten return to work in employees with fatigue symptoms and sick leave with a cognitive-behavioural treatment model [ 31].

## Clinicians Can Carry Out Psychological Interventions

A recent Cochrane review of psychological interventions carried out by general practitioners concluded that individual clinicians are able to incorporate such interventions into their treatment [ 32]. For example, Annette Blankenstein showed that a cognitive-behavioural treatment model for somatising patients could be applied by general practitioners after a 20-hour training programme [ 33]. In the other trial mentioned, occupational physicians also were able to carry out cognitive-behavioural treatment in patients with adjustment disorder after a brief training only [ 29].

The implication of these studies for clinical practice is firstly that all doctors should ask patients if they work and if they have reported sick. Possible hindrances for return to work such as a failure to make special arrangements in the workplace or misconceptions of disability should be explored. These issues can subsequently be addressed by referring patients to an occupational health professional or by using the cognitive-behavioural techniques mentioned above. To facilitate the implementation of these measures in practice, clinical guidelines should include guidance on return-to-work interventions.

## Abbreviations

RCT: randomised controlled trial
WHO: World Health Organization
